# Fatal Errors

**Published:** 1890-07

**Authors:** J. H. Jackson

**Affiliations:** Flint, Michigan


### FATAL ERRORS.

J. H. Jackson, M. D., Flint, Michigan.

To be able to repeat a train of symptoms and say to what remedy they belong is quite another matter from being able to recognize or elicit correctly the same symptoms when present in the sick.

One may be able to repeat symptoms by the hundred, and be far from proficient in the art of prescribing homœopathically.

Hahnemann has truly said that when the symptoms of the sick have been committed to writing, by far the most difficult part has been accomplished.

This statement is strictly true. The "*symptoms of the sick*" written down in the exact language of the patient, is by far the most difficult part of the art of healing. Not alone the symptoms that the patient voluntarily complains of, but also the particular symptoms that the adept in *eliciting* symptoms may bring out by judicious questioning, and in addition thereto the symptoms that the eye alone may discern.

So, not only must the exact langugage of the patient be recorded in writing, but also the language of each particular case of sickness, such as every visible peculiarity that may be discerned by the sense of sight, or any of the other senses.

To ask direct or leading questions is a fatal error.

To hastily select a remedy for a few or many symptoms that are complained of, without a complete examination of the history of all previous attacks of sickness, particulary in cases that have been treated by improper allopathic-law-of-metastasis-violating-treatment, is a fatal error.

To select a remedy however correctly for a group of symptoms that the patient may complain of, without *going back* to previous maltreated attacks of sickness, and hearing the peculiarities of such attacks, is a fatal error. The picture that the patient voluntarily complains of may be the totality of the *then* annoying symptoms, but such a picture is *not* the totality demanded by strict Hahnemannian Homœopathy.

The examination of the sick in a truly Hahnemannian manner is, indeed, a fine art.

The method of Hahnemann is the *scientific* method—that is the *knowing* method, not the *guessing* method, nor the I-think method, but the knowing method—and if there is one thing above another that we as true followers of an inspired master should ever keep uppermost in our minds, it is that nearly every case of chronic and acute sickness has its genesis in a violation of the law of metastasis by allopathic vital force perverting methods.

To illustrate: a man presented himself for treatment for a group of symptoms that are usually called dyspepsia. The symptoms were many, and not particularly covered by any remedy. A doctor styling himself a follower of Hahnemann had made sixty prescriptions, and finally abandoned the case with the comforting assurance that the patient was incurable, and would finally die of cancer of stomach.

An examination of the history of this case, elicited the fact that this man had been treated by injections for gonorrhœa some ten years before. Careful questioning made it plain to me that the character of the previous sickness demanded Nux-vomica, and upon the strength of the symptoms of *ten years ago*, $Nux^{mm}$ was given, with a return of the gonorrhœal discharge and a complete relief of all the distressing symptoms of the stomach, three months in all being necessary to restore the health of this "incurable" patient.

It would have been a very fatal error not to have looked up the past history of this man's ailments.

Another case in illustration. A man consulted me for fig-warts. There were large fig-warts on left side of corona glandis. The corona glandis and part of the glans had projections which looked exactly like the rough part of a cat's tongue.

Gonorrhœa had been suppressed about a year before consulting me. The same homœopathic (?) M. D. had treated this case, and I have no doubt every remedy recommended for fig-warts, that is, the "usual" remedies, had been given. But the fig-warts stood out in bold relief, so did something else, viz.: the *individuality of the patient.*

A careful examination unfolded the fact that the patient had had attacks of headache of a throbbing character that would come suddenly and leave as suddenly as they came, usually at three P. M., and often at three A. M. A very full pulse, prominence of blood-vessels in temporal region. Eyes sensitive to light.

Now, I never before had prescribed Belladonna for fig-warts, and had I been a "usual" remedy prescriber, I probably would never have prescribed it, but as it is a fatal error to give remedies *because* they are *usual* remedies, and as I try to avoid fatal errors, I gave $Belladonna^{cm}$, a dose once a week, and then once in three days, till I found that my patient needed it every night for a few weeks, and the result was a return of a watery gonorrhœal discharge, and a cure of fig-warts, headaches, and all symptoms complained of.

It is plainly a fatal error to ignore the individuality of our patients, and prescribe "usual" remedies that correspond with pathological lesions.

It should never be forgotten that improper medication locally applied is responsible for nearly all chronic ailments. In all chronic cases too much care cannot be expended in eliciting a correct picture of former attacks of sickness.

Many failures might be avoided by a constant practice of going back over the history of every sickness our patients may have had, and it is the purpose of this paper to burn into the souls of all who may read it that a *violation of the law of metastasis is responsible for nearly all chronic ailments, and the cause of the severity and obstinacy of many acute attacks of sickness.*

